# Estimating the incidence of breast cancer in Africa: a systematic review and meta-analysis

**DOI:** 10.7189/jogh.08.010419

**Published:** 2018-06

**Authors:** Davies Adeloye, Olaperi Y. Sowunmi, Wura Jacobs, Rotimi A David, Adeyemi A Adeosun, Ann O. Amuta, Sanjay Misra, Muktar Gadanya, Asa Auta, Michael O Harhay, Kit Yee Chan

**Affiliations:** 1 Nigerian Urban Reproductive Health Initiative, Abuja, Nigeria; 2 Johns Hopkins Centre for Communication Programs, Baltimore, Maryland, USA; 3 Centre for Global Health Research and the World Health Organization Collaborating Centre for Population Health Research and Training, Usher Institute, University of Edinburgh, Scontland, UK; 4 Computer and Information Sciences, Covenant University, Ota, Ogun State, Nigeria; 5 Department of Health Science, California State University, Fullerton, California, USA; 6 Department of Urology, Morriston Hospital, Abertawe Bro Morgannwg University Health Board, Swansea, UK; 7 Health Initiative Department, National Jewish Hospital, Denver, Colorado, USA; 8 Department of Health Studies, Texas Woman's University, Denton, Texas, USA; 9 Department of Computer Engineering, Atilim University, Turkey; 10 Department of Community Medicine, Aminu Kano Teaching Hospital, Bayero University, Kano, Nigeria; 11 School of Pharmacy and Biomedical Sciences, University of Central Lancashire, Fylde Road, Preston, UK; 12 Department of Biostatistics, Epidemiology and Informatics, Perelman School of Medicine, University of Pennsylvania, Philadelphia, Pennsylvania, USA; 13 Palliative and Advanced Illness Research (PAIR) Center, Perelman School of Medicine, University of Pennsylvania, Philadelphia, Pennsylvania, USA

## Abstract

**Background:**

Breast cancer is estimated to be the most common cancer worldwide. We sought to assemble publicly available data from Africa to provide estimates of the incidence of breast cancer on the continent.

**Methods:**

A systematic search of Medline, EMBASE, Global Health and African Journals Online (AJOL) was conducted. We included population- or hospital-based registry studies on breast cancer conducted in Africa, and providing estimates of the crude incidence of breast cancer among women. A random effects meta-analysis was employed to determine the pooled incidence of breast cancer across studies.

**Results:**

The literature search returned 4648 records, with 41 studies conducted across 54 study sites in 22 African countries selected. We observed important variations in reported cancer incidence between population- and hospital-based cancer registries. The overall pooled crude incidence of breast cancer from population-based registries was 24.5 per 100 000 person years (95% confidence interval (CI) 20.1-28.9). The incidence in North Africa was higher at 29.3 per 100 000 (95% CI 20.0-38.7) than Sub-Saharan Africa (SSA) at 22.4 per 100 000 (95% CI 17.2-28.0). In hospital-based registries, the overall pooled crude incidence rate was estimated at 23.6 per 100 000 (95% CI 18.5-28.7). SSA and Northern Africa had relatively comparable rates at 24.0 per 100 000 (95% CI 17.5-30.4) and 23.2 per 100 000 (95% CI 6.6-39.7), respectively. Across both registries, incidence rates increased considerably between 2000 and 2015.

**Conclusions:**

The available evidence suggests a growing incidence of breast cancer in Africa. The representativeness of these estimates is uncertain due to the paucity of data in several countries and calendar years, as well as inconsistency in data collation and quality across existing cancer registries.

Cancer is a leading cause of morbidity and mortality among women globally, accounting for about 17.5 million cancer cases and 9 million deaths in 2015 [1]. Breast cancer is now ranked the most common cancer worldwide, increasing from 1.7 million incident cases in 2005 to 2.4 million cases in 2015 [2]. Insights into the epidemiology and risks associated with breast cancer have seen relative improvements in the response to breast cancer across population groups, particularly in high-income settings [3]. However, in Africa and many low- and middle-income countries (LMICs), several challenges including poor health infrastructure, incomplete vital registrations, lack of population awareness, delayed health seeking behavior and low levels of female education and empowerment have led to high mortality from breast cancer [4], and also engendered a complex barrier to improving public health response to breast cancer in these settings [5].

The International Agency for Cancer Research (IARC) estimated that the incidence of breast cancer ranged from 27 per 100 000 women in central Africa to 39 per 100 000 women in southern Africa in 2012 [6]. Although, the incidence of breast cancer appears to be relatively low in Sub-Saharan Africa (SSA), survival from the disease is also generally poor in the region, with high mortality recorded in many settings [6,7]. The poor survival of breast cancer patients in SSA has been associated with late presentation, poor health care infrastructure and lack of adequate funding, amidst other competing public health challenges [5]. Moreover, in isolated SSA countries with relatively good breast cancer care services, inequity and unaffordability of the services provided have been widely reported [5,8]. Consequently, late presentations and advanced stages at diagnosis of several breast cancer cases have been prevalent, likely explaining the higher mortality rates reported [7]. With ageing, population growth, and adoption of unhealthy lifestyles, the burden of breast cancer is projected to double in Africa by 2030 [9], especially in the absence of effective public health policies and interventions [9]. See **Box 1** for an outline of what is already known, and what this study adds.

Box 1 **About the study**

**What is already known?**

The International Association of Cancer Registries (IACR) has been the main sources of cancer data in Africa, particularly by aiding African countries towards establishment of cancer registries. North Africa appears to have more robust data on cancer, with estimated incidence rates of breast cancer across North African states comparable to some high-income settings. In SSA, current reports reveal an increasing number of breast cancer cases diagnosed in the ages 35-49 years, with many presenting at advanced late-stage disease. Largely, data availability remains an ongoing limitation in understanding and estimating the incidence of breast cancer on the African continent.

**What this study adds**

This study provides the first systematic review and meta-analysis of publicly available evidence on the incidence of female breast cancer in Africa. There is limited data on cancer in Africa, particularly in Central Africa; only 22 African countries were included in this study. Population-based cancer registries remain the main sources of data on breast cancer in Africa as these provided 67% of all data points. Our estimated incidence of breast cancer increased between 2000 and 2015 across both registries, suggestive of a rising breast cancer incidence in Africa. The mean age of populations covered ranged from 30.6 to 60.8 years, with over 33% and 81% of population in ages 30-49 years, and 30-59 years, respectively.

Breast cancer prevention and control in Africa is relatively limited, owing in part to a lack of reliable epidemiologic risk factor data and information from which evidence-based interventions could have been developed [10,11]. For example, out of 46 World Health Organization (WHO) member states in SSA, only 20 (43.4%) have active cancer registries [12], spanning a wide range of coverage and completeness. These registries are poorly funded, largely limited to specific sub-national population groups (or even not population-based), and often do not meet standards required for active collation of cancer data [12]. Moreover, IARC also reported that due to lack of data on cancer incidence and mortality across developing countries, cancer estimates in these countries have been derived from frequency data or rates from neighboring countries with contextual similarities [9]. These efforts have no doubt contributed to the understanding of cancer burden in these settings [13]. However, intensifying ongoing efforts in the active collation of data from population- and hospital-based cancer registries across Africa and many developing countries may encourage further cancer-related research activities toward improving the overall representativeness of estimates provided [14,15]. Based on publicly available data, we estimated the incidence of breast cancer in Africa separately from population- and hospital-based cancer registries. We also examined the potential implication of the quality of data available from these registries on future responses to breast cancer in the region.

## METHODS

### Search strategy and data sources

We conducted an initial scoping literature search to identify Medical Subject Headings (MESH) and relevant keywords, following which a final search strategy was developed. We conducted a systematic search of Medline, EMBASE, Global Health and African Journals Online (AJOL), with search dates set from January 1980 to December 2016. We also conducted additional searches of Google Scholar, and websites of the International Association of Cancer Registries (IACR) and WHO African Region (AFRO). We equally reviewed the “*GLOBOCAN studies*” [6,9,16], “*Cancer Incidence in Five Continents (CI5) series*” [17], and “*Cancer in Africa: Epidemiology and Prevention*”[18] for more studies or additional data for studies already selected. Reference lists of initially selected studies were further hand-searched. The list of African countries was based on the World Bank list of economies [19] (**Table 1**).

**Table 1:** Search terms for studies on breast cancer in Africa

Number	Searches
**1**	africa/ or africa, northern/ or algeria/ or egypt/ or libya/ or morocco/ or africa, central/ or cameroon/ or central african republic/ or chad/ or congo/ or “democratic republic of the congo”/ or equatorial guinea/ or gabon/ or africa, eastern/ or burundi/ or djibouti/ or eritrea/ or ethiopia/ or kenya/ or rwanda/ or somalia/ or sudan/ or tanzania/ or uganda/ or africa, southern/ or angola/ or botswana/ or lesotho/ or malawi/ or mozambique/ or namibia/ or south africa/ or swaziland/ or zambia/ or zimbabwe/ or africa, western/ or benin/ or burkina faso/ or cape verde/ or cote d'ivoire/ or gambia/ or ghana/ or guinea/ or guinea-bissau/ or liberia/ or mali/ or mauritania/ or niger/ or nigeria/ or senegal/ or sierra leone/ or togo/
**2**	exp vital statistics/ or exp incidence/
**3**	(incidence* or prevalence* or morbidity or mortality).tw.
**4**	(disease adj3 burden).tw.
**5**	exp “cost of illness”/
**6**	exp quality-adjusted life years/
**7**	QALY.tw.
**8**	Disability adjusted life years.mp.
**9**	(initial adj2 burden).tw.
**10**	exp risk factors/
**11**	2 or 3 or 4 or 5 or 6 or 7 or 8 or 9 or 10
**12**	exp breast cancer/
**12**	1 and 11 and 12

### Selection criteria

We included population-based or hospital-based cancer registry studies on breast cancer conducted primarily on African population groups, and providing crude estimates of the cases or incidence of breast cancer among women in the population over a specified period. Studies were included if they were completed on or after the year 2000, to give a fair representation of the current burden. Hospital-based studies that only reported cases of breast cancer were also included if they provided sufficient information on the reference population to allow an estimation of the population or person-years at risk. We excluded studies on non-human subjects, and those that were mainly reviews, case reports, opinions or editorials. No language restrictions were applied.

### Confirmation of breast cancer diagnosis

We included studies that identified breast cancer based on histological diagnosis with or without *i)* clinical evaluation by a physician, *ii)* radiological investigations (mammography, breast ultrasound, computed tomography scan or magnetic resonance imaging), *iii)* laboratory tests (estrogen or progesterone receptor (ER or PR) status, the human epidermal growth factor receptor 2 (HER2) status, and breast cancer antigen (BRCA1 or BRCA2) or *iv)* community reported cases.

### Quality criteria

Each study was assessed for quality according to a set of five (5) predefined criteria. The first criterion was based on the cancer registration process. This considered how cancer registries collated data and the approach employed for data ascertainment. The second considered the coding criteria employed across studies to determine if the reported cancer types were classified according to the primary anatomic site (topography) or cellular characteristics (morphology—histology, behaviour, and grade) using the international classification of diseases (ICD) and oncology (ICD-O) guidelines [20-22]. The third assessed how population or person-years at risk were generated in each study. The fourth and fifth criteria checked whether the population covered in each study was representative of the target (subnational) and national populations, respectively. Each criterion was scored *one* (1), and studies were finally graded as *high* (4-5), *moderate* (2-3) or *low* (0-1) quality based on the number of criteria they met (Table S1 in **Online Supplementary Document(Online Supplementary Document)**). All low-quality studies were excluded from the review.

### Data extraction

Two reviewers (OYS and AA) independently screened studies against the inclusion and exclusion criteria and performed the data extraction. Any disagreement over article inclusion, exclusion or data extraction between the two initial reviewers was resolved through a final assessment by a third reviewer (DA). As we already employed a two-stage search and extraction process based on a combination of independent review and reassessment, we did not calculate Kappa statistics to determine agreement between the reviewers. We extracted data and relevant information systematically from each study. This included location, period, design, cancer registry, confirmation of diagnosis, data collation methods, coding criteria, data ascertainment and modality with which population or person years at risk were generated. The basic numerical data extracted were mean age (or age range), population or person years at risk, cancer cases, and crude incidence. For studies conducted on the same cancer registry over the same period, we selected the first chronologically published study, and supplementary data from other studies were added to the selected paper. All extracted data were separated into hospital-based and population-based studies, further sorted based on different regions in Africa (central, east, north, south, and west), and stored in Excel 2013 (Microsoft Inc, Redmond, WA, USA).

### Data analysis

From extracted crude incidence rates of breast cancer, we conducted a random effects meta-analysis (DerSimonian and Laird method) [23]. For studies not reporting population or person years at risk, we checked the UN population projections of the country for the study period and multiplied by the years covered. For sub-national populations, we applied the subnational population ratio to standardize the United Nations (UN) projections of the country for the study period. Standard errors were estimated from the crude incidence rates and person years assuming a Poisson distribution. Crude meta-estimates and confidence intervals (expressed per 100 000 person-years) were pooled from individual crude incidence rates and reported by registry-type for the African continent, the African sub-regions, age groups, and the years 2000 and 2015. I-squared (I^2^) statistics and subgroup (sensitivity) analysis were conducted to assess heterogeneity between studies. We conducted a meta-regression analysis (with age and year as confounders) to further check consistency of the data extracted and estimates reported for hospital-based and population-based registries, respectively. All statistical analyses were conducted on Stata 13.1 (Stata Corp LP, College Station, TX, USA).

## RESULTS

### Systematic review

**Figure 1:**
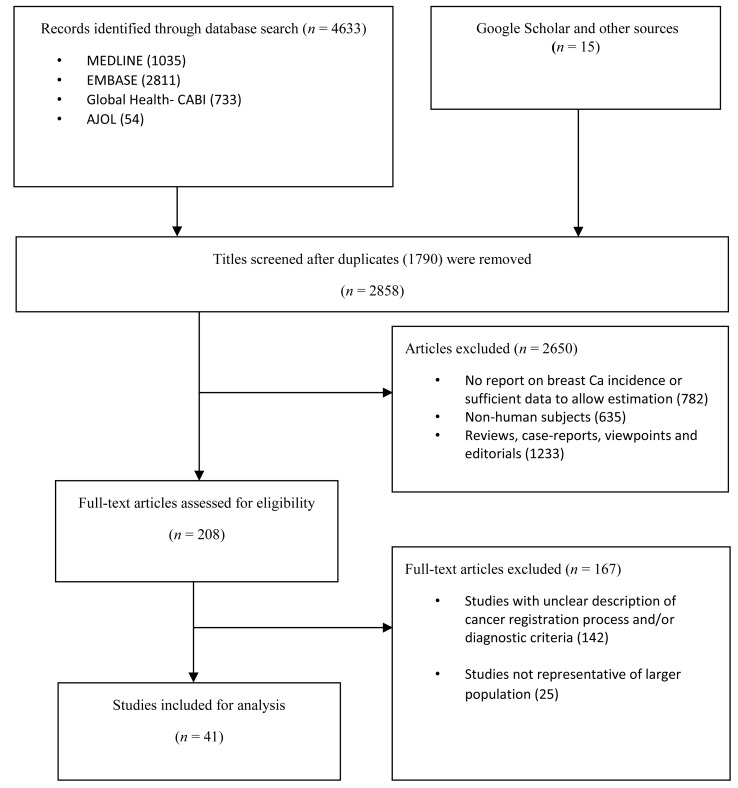
PRISMA flowchart of search strategy.

The literature search returned a total of 4648 studies. There were 4633 studies identified from the databases—Medline (1035), EMBASE (2811), Global Health (733) and AJOL (54). 15 additional studies were identified from Google Scholar, IARC and WHO AFRO websites, and hand-searching of reference lists. A total of 2858 records remained after duplicates were excluded. After reviewing titles for relevance (ie, studies providing incidence of breast cancer in any African location), 2650 studies were excluded, giving a total of 208 full texts that were assessed. After applying the selection criteria, 167 studies were excluded. A total of 41 studies were finally retained for the review [24-64] (**Figure 1**).

### Study characteristics

The 41 studies retained were conducted across 54 study sites (locations) in 22 African countries (**Table 2**). West Africa had the highest number of study sites (16) with seven of these sites located in Nigeria. South Africa closely followed with 6 sites while others were Uganda and Tunisia (5 sites each), Morocco (4 sites), Algeria, Ghana, and Libya (3 sites each), and Cameroon, Egypt, Mozambique and Niger (2 sites each). In total, SSA had 36 sites (70%) while North Africa had 18 sites (30%) (see **Table 2** for details). Population-based registries accounted for 67% of all study sites. These population-based registries reported mainly active or passive case findings across referral hospitals, pathology laboratories, and communities within the geographical remit of the cancer registry. Data were abstracted mostly from pre-designed pathology/laboratory forms confirming breast cancer diagnosis, with all sites reporting morphological/histological verification of the breast cancer cases. Mean age of study population ranged from 30.6 to 60.8 years (median 50.2 years), with over 33% in the age group 30-49 years, and over 81% in 30-59 years (**Table 2**).

**Table 2:** Characteristics of included studies

Author	Study period	Type of study	Cancer registry	Geographical remit of registry /centre	Confirmation of diagnosis	Coding criteria	How registry/centre collected data	Data ascertainment	How population or person-years at risk were generated	Who runs the registry/ center	Incidence rate/ 100 000*	Quality grading
Adewuyi et al. [24]	2004-2008	Retrospective study	Hospital-based records	Zaria, Nigeria	Physician diagnosis, Histologically verified	ICD-O	Collated from new patients laboratory results	Patients' folders were reviewed retrospectively with a structured pro-forma	Based on population of Zaria in 2004, standardized using UN population projections for women in Nigeria, multiplied by the years of the study	Ahmadu Bello University Teaching Hospital, Nigeria	9.04	Moderate
Bedwani et al. [25]	2001-2010	Population-based registry study	Alexandria Cancer Registry	Alexandria, Egypt	Histologically verified	ICD-0-2	Data collated from all public and private hospitals and pathology laboratories	Through laboratory notification forms	Based on census projections, age standardized rate calculated for 5 y age groups	Alexandria Cancer Registry	57.9	High
Bodalal et al. [26]	2012	Hospital-based registry study	Hospital-based cancer registry	Eastern Libya	Clinical, radiological, and histological diagnosis	ICD-O	Collated from oncology reports of patients in the hospital	Data were abstracted from pathology forms detailing cancer type	Based on Libyan census, taking into consideration the appropriate population growth.	Not reported	37.4	High
Bouchbika et al. [27]	2005-2007	Population-based registry survey	Greater Casablanca Cancer Registry	Casablanca, Morocco	Tumour site, Radiology, Histologically verified	ICD-O-3, converted to ICD 10 for tabulation	Collated from relevant health institutions across the region	Registry forms were filled for all medical records and pathology reports mentioning cancer	Based on National Institute of Statistics estimates of the growth rate of the Moroccan population in the period 2005-2007. Incidence rates calculated as described in the IARC Cancer in 5-continent-publications.	University Hospital of Casablanca	36.4	High
Bouchlaka et al. [28]	2003-2008	Hospital-based screening	Hospital-based	Ariana, Tunisia	Physician diagnosis, Mammography	Not reported	From patients screened	Radiological forms confirming cancer grade	Based on population of Ariana in 2003, standardized using UN population projections for Tunisia, multiplied by the years of the study to generate person years at risk	Family and Population National Office	4.9	Moderate
Calys-Tagoe et al. [29]	2012	Hospital-based registry study	Hospital cancer registry	Accra, Ghana	Physician diagnosis, Histologically verified	ICD-O using CanReg-5 software	Data actively collated on all cancer cases presenting to all departments/units of the hospital	Data abstracted from medical records and pathology forms	Based on catchment population for the year under review. Standardized using UN population estimates for women in Morocco	Korle Bu Teaching Hospital, Accra, Ghana	47.9	Moderate
Chbani et al. [30]	2004-2010	Restrospective Study	No registry (hospital records)	Fez Boulemane region, Morocco	Physician diagnosis, Laboratory test, Histologically verified	ICD-O	Collated from hospital laboratory reports	Data abstracted from pre-designed laboratory and pathology forms	Based on population of Morocco in 2004, standardized using UN population projections, multiplied by the years of the study to generate person years at risk	Department of Pathology in Hassan II University Hospital of Fes	59.8	Moderate
Chokunonga et al. [31]	1991-2010	Retrospective study	Zimbabwe National Cancer Registry	Harare, Zimbabwe	Histologically verified	ICD-O-2 converted to ICD 10 for tabulating	Data collated from all public and private hospitals and pathology laboratories	Through laboratory notification forms	Population at risk was estimated through linear interpolation and based on census data	Zimbabwean government	19.1 (18.6)	High
Dem et al. [32]	2001	Retrospective study	Institut Curie Registry	Dakar, Senegal	Histologically verified	ICD-O	Collated from hospital laboratory reports	Data abstracted from pre-designed laboratory and pathology forms	Based on population of Dakar in 2001, standardized using UN population projections for Senegal, multiplied by the years of the study to generate person years at risk	Institut Curie of Dakar	40.03	Moderate
Denewer et al. [33]	2010	Hospital-based screening	General Hospital, Mansoura University	Dakahlia province, Egypt	Physician diagnosis, Ultrasound, Laboratory test, Mammography	ICD-O	Data collected at Mansoura University	Radiological forms confirming cancer grade	Based on projected population of Dakahlia province in 2010	Oncology Center and General Hospital, Mansoura University	30.5	Moderate
El Fakir et al. [34]	2009- 2011	Retrospective evaluative study	Various health centres	Temara, Morocco	Physician diagnosis, Mammography	ICD	Process and performance indicators collected at the individual level from the various health structures	Data were abstracted from pathology forms confirming diagnosis	Based on population of Tamara in 2009, standardized using UN population projections for Morocco, multiplied by the years of the study to generate person years at risk	Not reported	2.5	Moderate
El Mistiri et al. [35]	2003-2005	Retrospective survey	Benghazi Cancer Registry	Northeastern Libya	Tumour site, Radiology, Histologically verified	ICD-O-3	Active searching of hospitals, public and private laboratories, and death certificates	Data were abstracted from pathology forms confirming diagnosis	Based on projected census population in Benghazi, standardized using UN population projections for Libya, multiplied by the years of the study to generate person years at risk	Garyounis University, Faculty of Medicine, National Research Centre building, Benghazi	23	High
El Mistiri et al. [36]	2003	Prospective survey	Benghazi Cancer Registry	Northeastern Libya	Tumour site, Radiology, Histologically verified	ICD-O-3	Active searching of hospitals, public and private laboratories, and death certificates	Data were abstracted from pathology forms confirming diagnosis	Based on 2003 population in Benghazi. Incidence reported for one year	Benghazi Cancer Registry	5.88	High
Enow Orock et al. [37]	2004-2011	Retrospective survey	The Yaounde Cancer Registry	Yaounde, Cameroon	Physician diagnosis, Mammography, Histologically verified	ICD-O	Collated from pathology laboratories within the region	Data abstracted from pre-designed laboratory and pathology forms	The 2010 population census data was used as a base for the population estimates to calculate the incidence rates for each year.	General Hospital, Yaounde	24.02	High
Garba et al. [38]	1992-2009	Retrospective and descriptive study	National Cancers Register of Niger Republic	Niger	Tumour site, Radiology, Histologically verified	ICD-O-2	Active searching of hospitals, public and private laboratories, and death certificates	Data were abstracted from pathology forms confirming diagnosis	Based on projected census population in Nigeria, standardized using UN population projections, multiplied by the years of the study	Not reported	20.2	High
Hamdi Cherif et al. [39]	1986-2010	Observational study	Cancer Registry of Setif (population-based)	Province of Setif, Algeria	Tumour site, Radiology, Histologically verified	ICD-O-3	Active searching of hospitals, public and private laboratories, and death certificates	Registration validity and completeness were evaluated using percentage of microscopic verification (MV) index	The corresponding population was obtained from the Algerian Institute of Statistics. Age-standardized rates (world population) (ASR-WR) were computed for the study calendar period	Cancer Registry of Setif, Tunisia	39.5	High
Jedy-Agba et al. [40]	2009-2010	Retrospective survey	Ibadan Population Based Cancer Registry (IBCR) and the Abuja Population Based Cancer Registry (ABCR)	Ibadan & Abuja, Nigeria	Tumour site, Radiology, Laboratory tests, Histologically verified	ICD-O-3	Active search of general and specialist hospitals, pathology laboratories and privately owned clinics and hospitals in Ibadan and Abuja	Data abstracted from notification forms onto CanReg4 software for storing, checking and processing data.	University College Hospital, Ibadan & National Hospital Abuja	Nigerian National cancer registry program	38.2 (25.8)	High
Korir et al. [41]	2004-2008	Retrospective survey	Nairobi Cancer Registry, a population-based cancer registry	Nairobi, Kenya	Tumour site, Radiology, Laboratory tests, Histologically verified	(ICD-0 3), converted to ICD-10 for tabulation	Active case finding from medical records departments, where disease index cards and patient-care registers were used to identify cancer cases	Relevant information on cancer cases was abstracted onto predesigned registration forms and entered into CanReg-5 software	Based on census population estimates for year 2004-2008. The average annual population of the registry area (Nairobi county) for the five year period was estimated.	Government, Nairobi cancer registry	17.2	High
Laryea et al. [42]	2012	Retrospective survey	The Kumasi Cancer Registry (population based)	Kumasi, Ghana	Histology, Clinical and Laboratory investigations	ICD-O-3	Data was from all clinical departments of the Komfo Anokye Teaching Hospital, Pathology Laboratory Results, Death Certificates and the Kumasi South Regional Hospital	Abstracted data was verified by a clinician and registry manager and transfered to CanReg 5 database.	Based on projected census population in Kumasi, standardized using UN population projections for Ghanaian women, multiplied by the years of the study	The Kumasi Cancer Registry	5.3	High
Lopes et al. [43]	2006-2014	Retrospective study	Angolan Institute of Cancer Control	Luanda, Angola	Physical breast examination, mammography, breast ultrasound, fine-needle aspiration (FNA)	Tumour, Node and Metastasis (TNM) 6th edition classification	Active collation of demographic, clinical and pathological information at diagnosis	Information on the number of breast cancer cases was reviewed and abstracted using standard forms	Based on UN population projections for Angola, multiplied by the years of the study	Angolan Institute of Cancer Control	31.7	Moderate
Lorenzoni et al. [44]	1991-2008	Retrospective study	Hospital registry	Maputo, Mozambique	Histology, Clinical and Laboratory investigations	ICD-O	Active collation of data from pathology department of the hospital	Data were entered into a Microsoft Access database from a predesigned pathology form	Based on censuses of the population of Mozambique in 1980, 1997 and 2007	Department of Pathology of the Maputo Central Hospital (MCH) started a cancer registry	26.2	High
Maalej et al. [45]	2004	Comparative clinical and epidemiological study	Hospital record	Tunisia	Histological diagnosis	ICD-O	Active censoring, analyses and collation of all cancers of the breast diagnosed in Tunisia across pathology laboratories in 2004	Data were abstracted from pathology notification forms following screening	Based on UN population projections for Tunisia for the year under review	Not reported	27.1	Moderate
Missaoui et al. [46]	2003-2006	Retrospective study	The population-based cancer registry of the centre of Tunisia	Sousse region, Tunisia	Histologically or cytologically verified	ICD-10	Proactive data collection from the pathology units of the public and private medical centres, and the departments of Radiotherapy, Oncology and Haematology of the University Hospital of Sousse	All the received data files are compared with cancer report lists. Registration validity and completeness were evaluated	Based on censuses of the total Tunisian population in 1994 and 2004. Crude incidence rates and five-year age-specific rates were calculated separately	Farhet Hached University Hospital, Sousse, Tunisia. Run by International agency of research on cancer, (IARC), Lyon, France.	28.3	High
Mohammed et al. [47]	1995-2004	Restrospective reviews	Kano cancer registry (KCR)	Kano, Nigeria	Histologically or cytologically verified	ICD-O	Records of cancer cases diagnosed based on histology or cytology and entered into the registry were retrieved	Data were abstracted from pre-designed pathology forms	Based on projected census population in Kano, standardized using UN population projections for Nigerian women, multiplied by the years of the study	Kano cancer registry	35.2	High
Msyamboza et al. [48]	2007-2010	Retrospective study	Nationwide cancer registry	Malawi	Clinically, histologically, or otherwise	ICD-O	New cancer cases registered from 2007 to 2010 were identified from hospital and clinic registers	Data were abstracted from standard pathology forms	Based on projected census population in Kano, standardized using UN population projections for Nigerian women, multiplied by the years of the study	Not reported	3.5	High
NCR South Africa [49]	2000-2011	National pathology-based study	National cancer Registry	South Africa	Histologically verified	ICD-O	Active collation of new cases of cancer from all public and private hospitals, laboratories and registries in the country	Data were abstracted from pre-designed pathology notification forms	Based on annual population. Crude and age standardized incidence reported annually	National cancer registry	27.2	High
Nayama et al. [50]	1992- 2000	Restropective and descriptive study	No registry, laboratory records	Niamey, Niger	Histological and laboratory investigations	ICD-O	Active finding of all cases of gynaecological and breast cancer across pathology laboratories in Niamey	Data were abstracted from pre-designed pathology forms	Based on projected census population in Niamey, standardized using UN population projections for Nigerien women, multiplied by the years of the study	Histopathologic laboratory of Niamey's Health faculty department, Niger.	47.8	Moderate
Nggada et al. [51]	2001-2005	Retrospective hospital study	University of Maiduguri Teaching Hospital Cancer Registry	Maiduguri, Nigeria	Histological and laboratory investigations	ICD-O	Active notification of histological verified cases of breast cancer in the hospital	Data were abstracted from pre-designed pathology forms	Based on projected census population in Maiduguri, standardized using UN population projections for Nigerian women, multiplied by the years of the study	University of Maiduguri Teaching Hospital, Nigeria	5.2	High
Nguefack et al. [52]	2006-2009	Prospective descriptive study	Hospital record Douala General Hospital	Douala, Cameroon	Clinical examination, ultrasonography, mammography, FNA, microbiopsy,auto examination	WHO classification	Active notification of histological verified cases of breast cancer in the hospital over the study period	Data were abstracted from pre-designed pathology forms	Based on projected census population in Douala, standardized using UN population projections for Cameroonian women, multiplied by the years of the study	Douala General Hospital	4.4	Moderate
Ntekim et al. [53]	2003-2006	Retrospective hospital study	Hospital records	Ibadan, Nigeria	Histological verification, Radiology, Physician diagnosis	ICD-O	Active reviews and collation of records of female patients with histologically confirmed breast cancer from 2003 to 2006	Data abstracted from pre-designed pathology notification forms confirming diagnosis	Based on population of Ibadan in 2003, standardized using UN population projections for women in Nigeria, multiplied by the years of the study	Radiotherapy Department of The University College Hospital, Ibadan Nigeria	51.0	Moderate
Ohene-Yeboah et al. [54]	2004-2009	Retrospective hospital study	Breast Care Center, Komfo Anokye Teaching Hospita	Kumasi, Ghana	Clinical, breast imaging (mammography or ultrasonography), tissue or pathologic diagnosis	Graded according to the modified Bloom-Richardson system.	Patients presenting with breast cancer at the hospital	Data were abstracted from a detailed proforma-based history of cancer cases	Based on projected census population in Kumasi, standardized using UN population projections for Ghanaian women, multiplied by the years of the study	Komfo Anokye Teaching Hospital in Kumasi, Ghana	57.4	Moderate
Pace et al. [55]	2015	Restrospective review	Butaro Cancer Center of Excellence, Rwanda	Rwanda	Histological diagnosis	ICD-O	Patients who presented with undiagnosed breast concern at the center	Clinical records and pathology forms confirming cancer diagnosis	Based on census population in Rwanda, standardized using UN population projections	Butaro Cancer Center of Excellence in Rwanda	2.96	Moderate
Parkin et al. [56]	1991-2006	Prospective study	Kampala Cancer Registry (population-based)	Kyadondo county, Kampala, Uganda	Tumour site, Morphology	ICD-O-2. Results converted to ICD-10 for tabulation	Data collected from several sources within Kyadondo county-screening pathology reports from government hospitals and private pathology laboratories; and conducting regular searches for cancer cases admitted or treated in hospitals.	Data abstracted from pre-designed pathology notification forms confirming diagnosis and then entered into CanReg software	Based on estimated annual population over the study period in Uganda. Incidence reported for 5-y age groups.	Makerere University College of Health Sciences, Uganda	24	High
Rahman et al. [57]	2003-2008	Retrospective study	Hospital records	Ilorin, Nigeria	Immuni-histochemical diagnosis	ICD-O	Patients with immunohistochemically confirmed breast cancer were reviewed and actively collated	Data were abstracted into into a proforma designed for the study	Based on census population in Ilorin, standardized using UN population projections for Nigerian women, multiplied by the years of the study	University of Ilorin Teaching Hospital, Nigeria	6.8	Moderate
Saeed et al. [58]	2009-2010	Retrospective study	National Population-based Cancer Registry	Khartoum, Sudan	Histological diagnosis, Radiology	ICD-10	The NCR staff used passive and active approaches to collect data on cancer diagnosed by all means in Khartoum State.	Data were abstracted from notification forms and entered into the computer using CanReg-5	Rates were age standardized to the 2010 Sudan Standard Population and 1966 and 2000 World Standard Population and expressed per 100 000 populations	Government-based registry	37.8	High
Sighoko et al. [59]	1998-2006	Retrospective study	National Population-based Cancer Registry	Gambia	Histological, radiological and laboratory diagnosis	ICD-O	Data were actively collated from public and private hospitals and pathology laboratories	Data abstracted from pre-designed pathology notification forms confirming diagnosis	The population at risk was derived from the national censuses. The mid-point of the study period was used to calculate incidence rates	Ministry of Health; Medical Research Council, Gambia	5.86	High
Somdyala et al. [60]	1998-2007	Retrospective survey	Population-based cancer registry	Eastern Cape Province, South Africa	Histological, radiological and laboratory diagnosis	ICD-O-3	Active and passive case finding of new cancer cases across health centres, district and referral hospitals and their laboratories	Data were manually abstracted from the records and predesigned notification forms	The 2001 census was used to estimate population at risk over the study period. Age standardized incidence rates reported	National cancer registry	7.6	High
Tonato Bagnan et al. [61]	2000-2008	Retrospective survey	Hospital records	Cotonou, Benin	Physician's diagnosis, Histological verification	ICD-O	Active reviews and collation of patient records with histologically confirmed breast cancer	Data were manually abstracted from the records and predesigned notification forms	Based on census population in Benin, standardized using UN population projections	Not reported	2.65	Moderate
Traore et al. [62]	2007-2009	Retrospective survey	Hospital Oncology Registry	Donka, Guinea	Physician's diagnosis, Histological verification	ICD	Active reviews and collation of patient records with histologically confirmed breast cancer	Data abstracted from pre-designed pathology notification forms confirming diagnosis	Based on census population in Conakry, standardized using UN population projections for Guinean women, multiplied by the years of the study	Unit of Surgical Oncology of Donka	10.99	Moderate
Wabinga et al. [63]	1991-2010	Prospective survey	Kampala Cancer Registry (KCR)	Kyadondo County, Kampala, Uganda	Tumour site, Morphology	ICD-O-2. Results converted to ICD-10 for tabulation	Active and passive case finding of new cancer cases across public and private hospitals and pathology laboratories	Data are abstracted onto notification forms, and are then entered into the registry database, using the CANREG system	The annual populations 2003-2010 were estimated assuming constant rates of change, within age-sex groups.	Kampala Cancer Registry (KCR)	31.2	High
Znati et al. [64]	2004-2009	Retrospective study	Hospital records	Fez, Morocco	Physician's diagnosis, Histological verification	ICD-O	Active reviews and collation of patient records with histologically confirmed breast cancer	Data abstracted from pre-designed pathology notification forms confirming diagnosis	Based on census population in Fez, standardized using UN population projections for Moroccan women, multiplied by the years of the study	Laboratoire d'anatomie pathologique, hôpital des spécialités, Fez, Maroc	0.43	Moderate

ICD – International Classification of Diseases; ICD-O – International Classification of Diseases and Oncology

*Last incidence rate included for multiple study periods.

**Figure 2:**
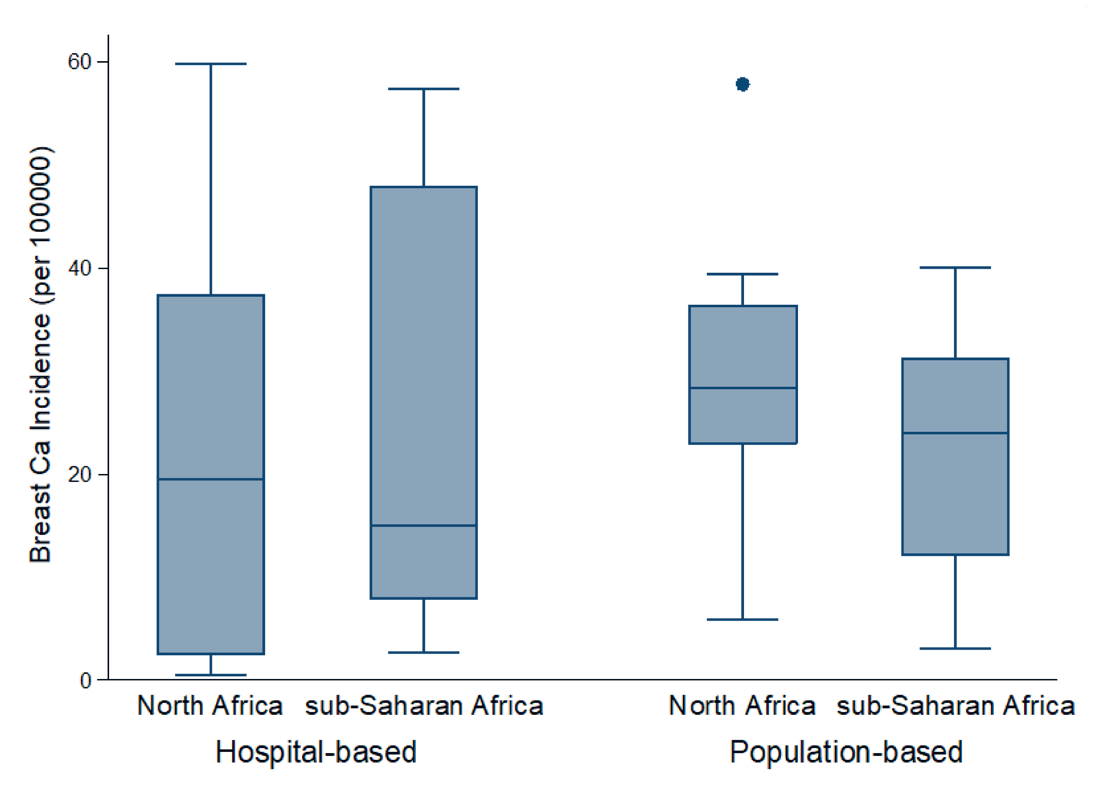
Distribution of crude incidence rates by registry type and African sub-region.

Following the quality assessment, 23 (56%) studies were graded high, of which (20) 87% were conducted in population-based registries, which suggests the internal consistency and validity of these registries. However, most hospital-based studies were of moderate quality, as 15 (83%) of the total 18 studies graded moderate were conducted in hospital-based registries (**Figure 2**, **Table 2**, Table S1 in **Online Supplementary Document(Online Supplementary Document)**). The basic limitations of many hospital-based registries were related to poorly defined data collation and ascertainment process. Moreover, catchment areas were also not well-defined, with this affecting the estimation of person-years across these registries.

### Pooled crude incidence rate of breast cancer in Africa

#### Population-based registries

**Figure 3:**
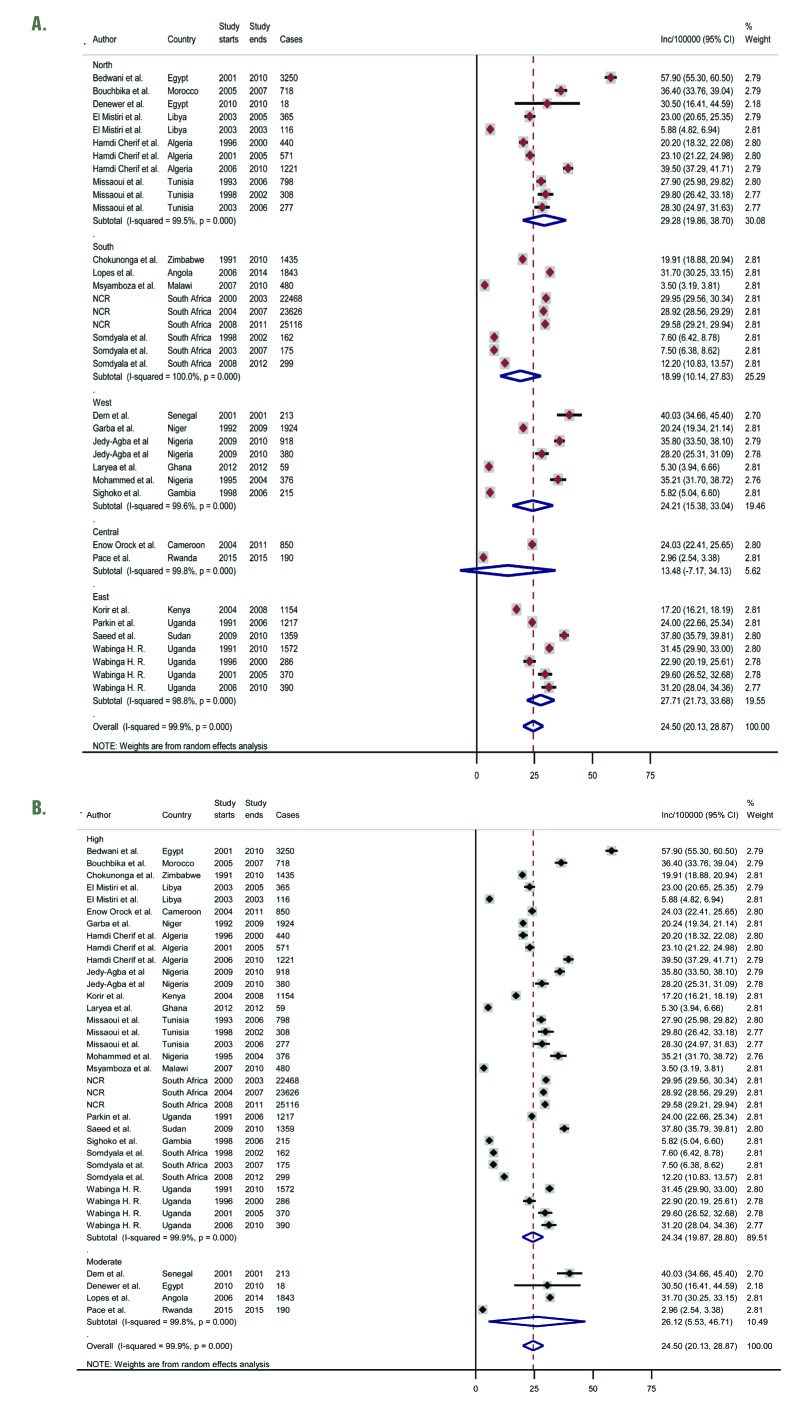
**A.** Pooled crude incidence rates of breast cancer in Africa by African region – population-based cancer registries. **B.** Pooled crude incidence rates of breast cancer in Africa by African region – population-based cancer registries (sensitivity analysis).

From population-based registries (36 study sites), the overall pooled crude incidence of breast cancer in Africa was 24.5 per 100 000 person years (95% Confidence Interval [CI]: 20.1-28.9) (**Figure 3**, plate A). The incidence rate in North Africa was higher at 29.3 per 100 000 (95% CI 20.0-38.7) than the pooled rates in SSA at 22.4 per 100 000 (95% CI 17.2-28.0). The estimated incidence rates in East Africa and West Africa were also relatively higher at 28.0 (95% CI 21.7-33.7) and 24.2 (95% CI 15.4-33.0) per 100 000, respectively. Southern Africa had a pooled rate of 19.0 (95% CI 10.1-27.8), while Central Africa had the lowest pooled incidence rates at 13.4 (95% CI 7.2-34.1) per 100 000. Due to high heterogeneity across studies (99.9%), we also reported the median incidence of breast cancer from all population-based registries, estimated at 28.1 per 100 000, with an interquartile range (IQR) of 18.6-31.3 per 100 000 (**Table 3**, **Figure 2**). Besides, a sensitivity analysis based on quality criteria showed studies graded as high quality had almost similar pooled incidence as the overall pooled incidence at 24.3 per 100 000 person years (95% CI 19.9-28.8), compared to moderate quality studies at 26.1 per 100 000 (95% CI 5.5-46.7) (**Figure 3**, plate B).

**Table 3:** Sub-group meta-analysis of breast cancer crude incidence rates in Africa

Headings	Data	Population-based registry			Hospital-based registry		
**Incidence per 100 000 (95% CI)**	**I^2^ (*P*-value)**	**Data points**	**Incidence per 100 000 (95% CI)**	**I^2^ (*P*-value)**	**Data points**
All	All	24.5 (20.1-29.0); *high quality* 24.3 (19.9-28.8)	99.9% (*P* <0.001)	36	23.6 (18.5-28.7); *high quality* 17.4 (2.2-32.5)	99.7% (*P* <0.001)	18
African region	Sub-Saharan Africa (SSA)	22.4 (17.2-28.0)	99.9% (*P* <0.001)	25	24.0 (17.5-30.4)	99.3% (*P* <0.001)	12
	Central Africa	13.4 (7.2-34.1)	99.8% (*P* <0.001)	2	4.4 (3.1-5.7)	–	1
	East Africa	28.0 (21.7-33.7)	98.8% (*P* <0.001)	7	-	–	-
	Southern Africa	19.0 (10.1-27.8)	100% (*P* <0.001)	9	19.4 (6.3-32.5)	95.7% (*P* <0.001)	2
	West Africa	24.2 (15.4-33.0)	99.6% (*P* <0.001)	7	27.4 (18.6-36.2)	99.4% (*P* <0.001)	9
	North Africa	29.3 (20.0-38.7)	99.5% (*P* <0.001)	11	23.2 (17.0-29.5)	99.9% (*P* <0.001)	6
Age	30-39	3.3 (2.7-3.8)	98.7% (*P* <0.001)	2	23.3 (17.0-29.5)	99.7% (*P* <0.001)	4
	40-49	22.4 (13.1-31.7)	99.6% (*P* <0.001)	7	7.1 (4.6-9.6)	91.3% (*P* <0.001)	5
	50-59	22.6 (18.6-26.6)	99.7% (*P* <0.001)	19	34.3 (17.6-51.0)	99.5% (*P* <0.001)	7
	60+	36.6 (30.7-42.4)	75.6% (*P* = 0.043)	8	27.1 (7.3-47.0)	97.9% (*P* <0.001)	2
Study period*	2000-2010	23.1 (18.7-27.5); *North Africa* 24.3 (16.1-32.5); *SSA* 22.6 (14.6-30.5)	99.7% (*P* <0.001)	20	19.7 (15.2-24.2); *North Africa* 13.1 (4.6-30.8); *SSA* 21.9 (15.4-28.3)	99.6% (*P* <0.001)	14
	2010-2015	26.3 (18.8-33.7); *North Africa* 43.6 (28.3-58.9); *SSA* 22.3 (16.7-27.9)	99.9% (*P* <0.001)	16	36.9 (4.1-69.6); *North Africa* 33.2 (6.7-73.1); *SSA* 47.9 (41.2-54.6)	99.7% (*P* <0.001)	4
Median (Interquartile range)		28.1 (18.6-31.3)	–	36	15.0 (5.0-47.9)	–	18

CI – confidence interval

*Indicates period when study ended.

Moreover, in the population-based registries, we observed an increasing incidence with increasing age of subjects and over the study period covered (**Table 3**). The pooled incidence rate was highest among persons aged 60 and above at 36.6 per 100 000 (95% CI 30.7-42.4) compared to an incidence rate of 3.3 per 100 000 (95% CI 2.7-3.8) among persons aged 30-39 years (**Table 3**). Between 2000 and 2015, the pooled incidence rate increased from 23.1 (95% CI 18.7-27.5) to 26.3 (95% CI 18.8-33.7) per 100 000, respectively (**Table 3**). Further analysis by region revealed incidence of breast cancer in North Africa increased from 24.3 per 100 000 (95% CI 16.1-32.5) to 43.6 per 100 000 (95% CI 28.3-58.9) between 2000 and 2015. However, the incidence was about the same over the same period in SSA at 22.6 per 100 000 (95% CI 14.6-30.5) and 22.3 per 100 000 (95% CI 16.7 -27.9) (**Table 3**).

The meta-regression analysis showed a significant association of the crude incidence rates with age and year (*P* = 0.0012) (**Table 4**), which possibly demonstrates a relative consistency and representativeness of the data captured by population-based cancer registries. Assuming our pooled population-based registry estimates accounted for population growth, ageing and other demographic and epidemiological changes in Africa, these rates would account for 93 890 (95% CI 76 007-111,774) breast cancer cases among women in 2000. This increased to 150 526 breast cancer cases by 2015 (95% CI 107 600-192,880), based on the UN demographic projections for Africa.

**Table 4:** Meta-regression analysis by registry type

Breast Cancer Incidence	Coef.	Std. err.	t	*P* > t	Upper CI	Lower CI
Population-based*						
Age	8.718566	2.188229	3.98	<0.001	4.26658	13.17055
Year	3.975006	3.495557	1.14	0.264	-3.136759	11.08677
_cons	6.098437	4.850205	1.26	0.217	-3.769378	15.96625
**Hospital-based†**						
Age	1.865169	6.001334	0.31	0.760	-10.92637	14.65671
Year	14.49101	13.73779	1.05	0.308	-14.79038	43.77241
_cons	-10.83332	27.9259	-0.39	0.704	-70.35596	48.68932

CI – confidence interval

*Number of observations = 36. REML estimate of between-study variance (tau2) = 105.9. % residual variation due to heterogeneity (I-squared_res) = 99.56%. Proportion of between-study variance explained (Adj R-squared) = 29.96%. Joint test for all covariates Model F(2,33) = 8.34. With Knapp-Hartung modification Prob>F = 0.0012.

Number of observations = 18

†REML estimate of between-study variance (tau2) = 443.7. % residual variation due to heterogeneity (I-squared_res) = 99.43%. Proportion of between-study variance explained (Adj R-squared) = 0.09%. Joint test for all covariates Model F(2,15) = 1.00. With Knapp-Hartung modification Prob>F = 0.3926.

#### Hospital-based registries

**Figure 4:**
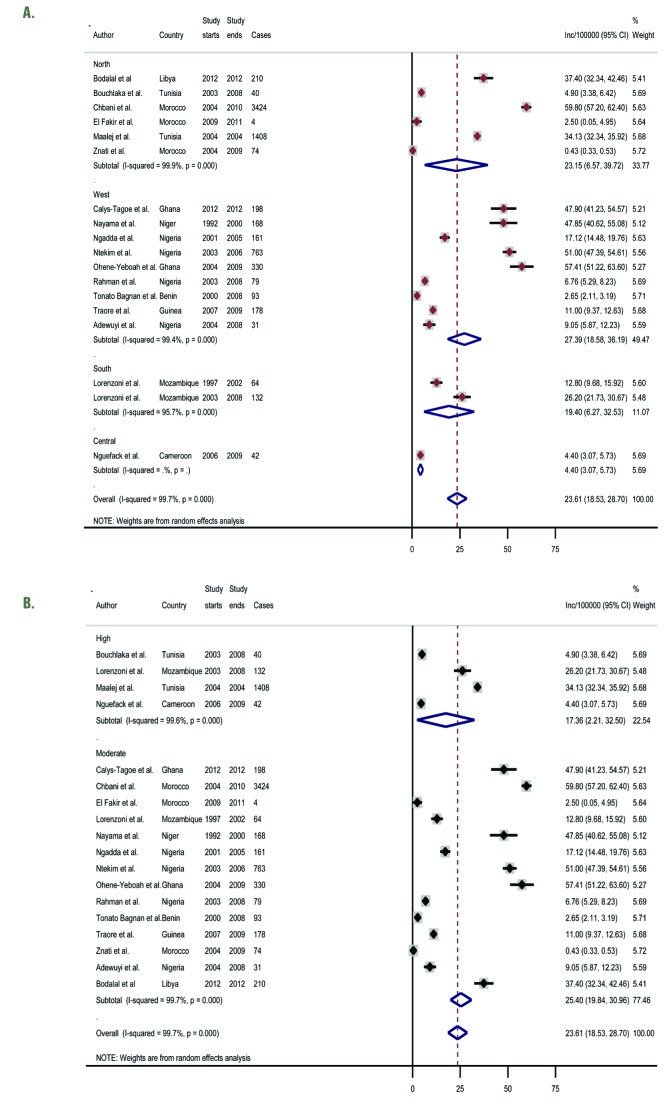
**A.** Pooled crude incidence rates of breast cancer in Africa by African region – hospital-based cancer registries. **B.** Pooled crude incidence rates of breast cancer in Africa by African region – population-based cancer registries (sensitivity analysis).

The results from the hospital-based registries (18 study sites) were slightly different (from the population-registries (**Figure 4**, plate A; **Table 3**). The overall pooled crude incidence rate was estimated at 23.6 per 100 000 (95% CI 18.5-28.7), with a median incidence of 15.0 per 100 000 (IQR: 5.0-47.9). Further sensitivity analysis showed high quality studies had a pooled incidence rate at 17.4 per 100 000 (95% CI 2.2-32.5) compared to 25.4 per 100 000 (95% CI 19.8-31.0) for moderate quality hospital-based studies (**Figure 4**, plate B).

In contrast to the regional pattern observed in the population-based registries, SSA and Northern Africa had quite similar pooled rates at 24.0 per 100 000 (95% CI 17.5-30.4) and 23.2 per 100 000 (95% CI 6.6-39.7), respectively. Central Africa also had the lowest incidence rate at 4.4 per 100 000 (95% CI 3.1-5.7) (**Figure 4**, plate A). Although pooled incidence rates increased from 19.7 per 100 000 (95% CI 15.2-24.2) to 36.9 per 100 000 (95% CI 4.1-69.6) between 2000 and 2015, this increase in incidence rates was not associated with advancing age (**Table 3**). Further analysis by region over the same period (2000-2015) showed that North Africa and SSA markedly increased from 13.1 per 100 000 (95% CI 4.6-30.8) to 33.2 per 100 000 (95% CI 6.7-73.1), and 21.9 per 100 000 (95% CI 15.4-28.3) to 47.9 per 100 000 (95% CI 41.2-54.6), respectively. However, incidence rates decreased sharply between ages 30-39 years and 40-49 years from 23.3 (95% CI 17.0-29.5) to 7.1 (95% CI 4.6-9.6) per 100 000; peaked at 50-59 years (34.3 per 100 000, 95% CI 17.6-51.0), but dropped again at age 60 years and above to 27.1 per 100 000 (95% CI 7.3-47.0) (**Table 3**). The meta-regression showed no significant relationship between incidence, age and years covered (*P* = 0.3926) (**Table 4**), which perhaps reflects incomplete data capture in the hospital registries (no data from East Africa), and is probably partly due to our inability to appropriately define the catchment area of the hospital.

## DISCUSSION

As the leading cause of cancer globally among women, breast cancer has continued to attract interest among experts and clinicians, particularly towards appropriately quantifying its burden and identifying risks across countries. In Africa, experts have continued to work towards addressing several challenges in the response to a growing cancer burden [14]. However, with relatively scant, incomplete, or poorly representative data on cancer in many settings, it remains difficult to effectively address this burden [15]. This study provides the first systematic review and meta-analysis of publicly available evidence on the incidence of female breast cancer in Africa with a view to improving the understanding of the epidemiology of the disease in the region. This endeavor yielded some findings.

Our study showed that population-based cancer registries remain the main sources of data on breast cancer in Africa as these provided 67% of data in this study. Current data from most hospital-based cancer registries are rather too incomplete and inconsistent to be deployed in the understanding of cancer epidemiology in Africa [12], as evidenced from the quality assessment. In the population-based registries, we observed that the estimated regional variation in breast cancer incidence in Africa was consistent with the GLOBOCAN 2008 and 2012 studies, with the incidence in North Africa higher than observed in SSA [6,9]. The estimated incidence of breast cancer in North Africa (29.3 per 100 000) was almost same as values reported by the global burden of diseases (GBD) collaborators for the region (29.7 per 100 000) [2]. However, the GBD collaborators noted a relatively higher incidence rate in SSA in 2015 at 34.1 per 100 000 compared to our estimate of 22.4 per 100 000 (95% CI 17.2-28.0) for SSA [2]. The GBD estimates are thus suggestive of a higher incidence of breast cancer in SSA compared to North Africa, which are in contrast to our findings of the opposite, and possibly raises potential questions regarding the rising trend of breast cancer in SSA compared to North Africa. The differences may be partly attributable to sources and completeness of data, as well as modeling approaches employed in the GBD estimates compared to the present study. Although some previous reports have shown that the estimated incidence rates of breast cancer across North African states are higher than in SSA, and comparable to rates in some high-income settings [65-67]. The regional differences in Africa may be indicative of racial and ethnic disparities in invasive breast cancer epidemiology in the region, as already observed in the variations in incidence and mortality patterns between black and white women globally [68,69]. Dickens and colleagues noted that the clinical spectrum of breast cancer in Africa is highly heterogenous across population groups [70], with this often linked to variations in age at diagnosis, staging, time trends, and genetic and environmental risks [69,71]. Moreover, the different stages of fertility transition currently observed in Africa as evidenced by declining births and widening birth intervals across population groups may also be an important driver of the regional differences [71].

Across both population- and hospital-based registries, we reported lower incidence rates in Central Africa. However, these estimates have wide uncertainty intervals, possibly reflecting the limited data in the region, and the substantial variation and imprecision in reported figures already noted across African countries [10,72]. Some authors have reported the lack of data in Central Africa as a major factor mitigating against the estimation of disease burden and ensuring evidence-based interventions in the region [12,73,74]. Balekouzou and colleagues specifically noted that the epidemiology of breast cancer in Central Africa remain poorly understood, owing to sparse data on cancer in the region [75]. Ongoing civil unrest in some parts of Central Africa influencing the capacity of the health systems to adequately capture cancer cases may have resulted in a relatively low cancer ascertainment.

The mean age of populations covered in both the population- and hospital-based registries ranged from 30.6 to 60.8 years (median 50.2 years), with over 33% and 81% of population in ages 30-49 years, and 30-59 years, respectively. This, albeit subject to further validations, may be suggestive of a high incidence of breast cancer among younger age groups in Africa. For example, Jedy-Agba and colleagues reported that many cases of breast cancer in SSA were diagnosed in the ages 35-49 years, with many presenting at advanced late-stage cancer [7]. Some authors have argued that the higher proportion of younger women in Africa, due to a relatively lower life expectancy on the continent, may have been responsible for the increasing breast cancer incidence reported among younger age groups [14,76]. However, epidemiological transitions, rapid urbanization with increased adoption of unhealthy lifestyles, increasing prevalence of obesity in younger populations, changing reproductive behaviours, including early menarche, low parity, advanced age at first pregnancy, and low self-breast examination for breast cancer among adolescents and young women have been identified as possible risk factors [14,76,77].

The poor state of many health systems in Africa and their declining capacity to lead cancer preventive initiatives and response to overall health needs of the population, as compared to developed countries, are also major concerns. Abdulrahman and Rahman reported that there are significant differences in age and stage at presentation between women in Africa and Europe, with more than half of breast cancer patients in Nigeria and Libya presenting at relatively younger age, mostly with advanced stage III or IV disease [78]. Parkin et al. equally noted that the lifetime risk of dying from breast cancer in young African women is about twice the risk in high-income countries [56].

Meanwhile, we observed some important variations in the estimates reported from population-and hospital-based registries in this study. Although our estimated incidence of breast cancer increased between 2000 and 2015 in both registries, suggestive of a rising breast cancer incidence in Africa, there was however a more significant rise in breast cancer incidence with advancing age in the population-based registries. This was clearly different from our finding from the hospital-based studies. Moreover, from population-based registries, the breast cancer incidence in North Africa (29.7 per 100 000) was higher than the incidence in SSA (22.4 per 100 000), while hospital-based registries provided relatively similar estimates (23.2 per 100 000 vs 24.0 per 100000). These variations, notwithstanding our estimation approach, further raise concerns on the completeness and representativeness of data in African registries. Population-based cancer registries have been widely regarded as being the gold standard for accurate data collation on cancer [79], as they cover wide geographical areas including communities, and employ standard coding methods and estimation of person-years [12]. However, challenges have been identified in the establishment and effective functioning of population-based registries across Africa due to poor funding, lack of skilled personnel, and low priorities from many governments, with this affecting the estimates provided [12,79]. Some authors have reported that hospital-based registries can indeed complement the few population-based registries, particularly by providing data on health service provision and access, which is useful for appropriate planning and public health response [79]. To address challenges with data consistency and representativeness, efforts may also be directed at improving geographical mapping of hospitals to appropriately determine the catchment area or population covered, and generate standardized methods to calculate cancer incidence that are representative of the population [12].

The application of precision medicine across world regions is still evolving [3]. However, translating this into prevention and management of breast cancer in Africa also requires additional efforts with cancer registration [3]. It is important for future research to decipher if the advanced stage of breast cancer presentation in Africa could be linked to unique aggressive biological characteristics of the malignancy, or some specific intrinsic host factors in African populations [7,76]. This is essential to guide health care practitioners to adequately respond to known and prevailing health disparities with respect to genetic determinants, predisposition and susceptibility to breast cancer across different ethnic groups on the continent [3]. However, the research capacity in this regard is relatively poor, and additionally limited by the lack of comprehensive and nationally representative data on breast cancer across African countries [5]. Hence, public health promoting behaviors that decrease breast cancer incidence still appears be most important and feasible preventive strategy across Africa.

The limitations of this study are also related to our key findings. First, though we performed a compressive data retrieval process, the empirical estimates we provided are limited by the absence of population-based cancer registries in many African countries (only 22 African countries represented), which has implications for the precision and representativeness of our estimates. Although, about 67% of studies were conducted in population-based cancer registries, many of these registries only covered subnational populations. As highlighted in the results, Nigeria and South Africa had the highest data points in this study. However, of all entries, South Africa particularly provided a complete national registry report of histologically-diagnosed breast cancer cases covering a period of 18 years, although only recent reports suggested an upgrade to population-based cancer registration system [80]. As noted, across data from hospital-based reports, we relied on the UN demographics for the country to estimate reference populations and person-years when they were not provided. This could have led to a relative under-estimation of cancer incidence due to resultant large denominators derived from the UN demographics. Besides, estimates were not age-standardized which limits direct comparisons with other world regions. Moreover, the heterogeneity across selected studies was high (I^2^>90%), which further reflects the need to address varying study designs, case ascertainments, referral approaches, and standards for collation of cancer data across different population groups, as these are important sources of heterogeneity. Despite these limitations, it is still important to present to the public the challenges arising from data availability on breast cancer research in Africa, the varying estimates available, and the implication of this on the response to a rising burden of breast cancer on the continent.

## CONCLUSIONS

The efforts of various researchers and organizations, particularly the IARC, United States National Cancer Institute (NCI) and African Organization for Research and Training in Cancer (AORTIC), must be acknowledged in the response to cancer in Africa. Data availability remains an ongoing limitation in understanding and estimating the incidence of breast cancer on the continent. Further issues from available data include inconsistency in data collation and quality across existing cancer registries. However, available evidence is suggestive of a growing incidence of breast cancer in Africa. With population growth and ageing, exposures to known risks, and relative weak health systems in Africa, it is unlikely this trend will change in the short-term in the absence of effective interventions, with this further straining the health system in many African countries. As various funders and institutions develop their focuses, we assert that strategies for controlling and treating breast cancer patients must include activities that support active and comprehensive data collation and cancer registration (population- and hospital-based) on the African continent, and more emphasis on the biological and histological characteristics of breast cancer cells across different population groups.
