# Supplementary material for: Estimating the incidence of breast cancer in Africa: a systematic review and meta-analysis
Source: J Glob Health. 2018 Apr 16;8(1):010419. doi: 10.7189/jogh.08.010419 (PMC5903682; doi:10.7189/jogh.08.010419)
Supplement: Online Supplementary Document [file jogh-08-010419-s001.pdf]

Online Supplementary Document

Adeloye et al. Estimating the incidence of breast cancer in Africa: a systematic review and meta-analysis

J Glob Health 2018;8:010417

Table S1: Quality assessment and grading

Author	Reg process	Cancer classification	Person-years calculation	Representative of target/subnational population	Representative of national population	Total score	Quality grading
Adewuyi et al. [24]	0	1	0	1	0	2	Moderate
Bedwani et al. [25]	1	1	1	1	0	4	High
Bodalal et al. [26]	1	1	1	1	0	4	High
Bouchbika et al. [27]	1	1	1	1	1	5	High
Bouchlaka et al. [28]	0	0	1	1	0	2	Moderate
Calys-Tagoe et al. [29]	1	1	0	1	0	3	Moderate
Chbani et al. [30]	0	1	1	1	0	3	Moderate
Chokunonga et al. [31]	1	1	1	1	0	4	High
Dem et al. [32]	1	1	0	1	0	3	Moderate
Denewer et al. [33]	0	1	1	0	0	2	Moderate
El Fakir et al. [34]	0	1	1	0	0	2	Moderate
El Mistiri et al. [35]	1	1	1	1	0	4	High
El Mistiri et al. [36]	1	1	1	1	0	4	High
Enow Orock et al. [37]	1	1	1	1	0	4	High
Garba et al. [38]	1	1	1	1	1	5	High
Hamdi Cherif et al. [39]	1	1	1	1	0	4	High

Jedy-Agba et al. [40]	1	1	1	1	0	4	High
Korir et al. [41]	1	1	1	1	0	4	High
Laryea et al. [42]	1	1	1	1	0	4	High
Lopes et al. [43]	0	1	1	1	0	3	Moderate
Lorenzoni et al. [44]	1	1	1	1	0	4	High
Maalej et al. [45]	0	1	1	1	0	3	Moderate
Missaoui et al. [46]	1	1	1	1	0	4	High
Mohammed et al. [47]	1	1	1	1	0	4	High
Msyamboza et al. [48]	1	1	1	1	1	5	High
Nayama et al. [49]	0	1	1	1	0	3	Moderate
NCR South Africa [50]	1	1	1	1	1	5	High
Nggada et al. [51]	1	1	1	1	0	4	High
Nguefack et al. [52]	0	1	1	0	0	2	Moderate
Ntekim et al. [53]	0	1	1	0	0	2	Moderate
Ohene-Yeboah et al. [54]	0	1	1	1	0	3	Moderate
Pace et al. [55]	0	1	1	1	0	3	Moderate
Parkin et al. [56]	1	1	1	1	0	4	High
Rahman et al. [57]	0	1	1	0	0	2	Moderate
Saeed et al. [58]	1	1	1	1	0	4	High
Sighoko et al. [59]	1	1	1	1	1	5	High
Somdyala et al. [60]	1	1	1	1	0	4	High
Tonato Bagnan et al. [61]	0	1	1	0	0	2	Moderate
Traore et al. [62]	0	1	1	0	0	2	Moderate
Wabinga et al. [63]	1	1	1	1	0	4	High
Znati et al. [64]	0	1	1	0	0	2	Moderate

Key
High (4-5)

Moderate (2-3)
